# Atypical Presentation of Hirschsprung's Disease in an Infant: Challenges in Early Diagnosis and Management of Recurrent Enterocolitis

**DOI:** 10.7759/cureus.73862

**Published:** 2024-11-17

**Authors:** Peter J Rogu, Emily Colalillo, Nicholas Rogu, George Rogu

**Affiliations:** 1 Medicine, New York Institute of Technology (NYIT) College of Osteopathic Medicine, Old Westbury, USA; 2 Pediatric Medicine, New York Institute of Technology (NYIT) College of Osteopathic Medicine, Old Westbury, USA

**Keywords:** diarrhea, enterocolitis, high-risk newborn, hirschsprung-associated enterocolitis, hirschsprung's disease, newborn jaundice, short track hirschsprung's disease

## Abstract

Hirschsprung's disease is a congenital condition characterized by the absence of ganglion cells in the intestines, leading to bowel obstruction. The lack of ganglion cells disrupts the normal motility of the intestines, causing a functional obstruction. This can lead to enterocolitis, an inflammation of the intestines, which is a serious complication in infants with Hirschsprung's disease. This case report follows a male infant with multiple admissions for recurrent enterocolitis secondary to undiagnosed Hirschsprung's disease with unresolving diarrhea. This study underscores the significance of early diagnosis and management, even in cases of atypical presentation, emphasizing the role of antibiotics, bowel decompression, and surgical intervention. The findings highlight the complexity of managing Hirschsprung's disease and offer insights into tailored approaches for optimizing patient outcomes.

## Introduction

Hirschsprung's disease (HD), also referred to as congenital aganglionic megacolon, is a developmental anomaly affecting the enteric nervous system, characterized by the absence of ganglion cells in the distal bowel, leading to functional obstruction [1]. This condition, diagnosed in approximately one in 5,000 live births and exhibiting a male predominance, stems from incomplete migration of neural crest cells during embryonic development, resulting in the absence of these cells in the myenteric and submucosal plexuses of affected bowel segments [1].

In individuals with HD, the absence of these cells disrupts normal peristalsis in the affected bowel segment, resulting in functional obstruction and subsequent dilation proximal to the aganglionic segment. The pathogenesis involves genetic factors, including mutations in genes such as *RET*, *EDNRB*, and *GDNF*, which are pivotal in the development of the enteric nervous system [2].

The clinical presentation of HD typically manifests shortly after birth in neonates or infants, characterized by symptoms of bowel obstruction such as constipation, abdominal distension, and failure to pass meconium within the first 48 hours of life [1]. Older children may present with chronic constipation, abdominal pain, and growth retardation due to chronic malnutrition. The severity and presentation of symptoms vary based on the length and location of the aganglionic segment.

Most cases of HD are diagnosed in the neonatal period or early infancy, with symptoms appearing soon after birth [3]. However, in cases where the aganglionic segment is shorter, diagnosis may be delayed until later childhood or adolescence, particularly when symptoms of chronic constipation or enterocolitis become more pronounced [3].

Enterocolitis occurs when the dilated, aganglionic segment of the bowel becomes inflamed and infected. This inflammation can lead to a range of symptoms, including fever, abdominal distension, explosive diarrhea (often containing blood), vomiting, lethargy, and poor feeding [4]. While not every child with HD will develop enterocolitis, it is estimated to occur in approximately 20%-30% of patients with HD, particularly in those with longer segments of aganglionosis or more extensive disease involvement [4,5].

Early recognition and prompt treatment of enterocolitis are crucial to prevent serious complications such as sepsis and bowel perforation. Management typically involves intravenous fluids, antibiotics targeted against enteric pathogens, bowel rest, and close monitoring in a hospital setting [5,6].

Diagnosing HD often involves different approaches, such as radiographs, manometry, and histology. Radiological evaluation through contrast enemas plays a crucial role in this process. This imaging technique helps differentiate HD from other causes of lower gastrointestinal obstruction and assists in estimating the length of the aganglionic segment [7]. Histological examination remains the definitive method for diagnosing HD. Various techniques for rectal biopsy are employed, including suction biopsy, punch biopsy, and open biopsy. The presence of a single ganglion cell in the specimen is sufficient to rule out HD [7]. The diagnosis of HD almost exclusively demands surgical intervention.

## Case presentation

The patient is a male infant, born at 39 weeks and two days gestation via c-section, who was admitted to the NICU immediately after birth due to suspected neonatal sepsis following a fever of 101.4°F. Mom was GBS (Group B Streptococcus)-positive and received appropriate penicillin treatment prior to delivery. Immediate treatment for the baby included antibiotics (ampicillin and gentamicin), ordering blood cultures, complete blood count (CBC), C-reactive protein (CRP), and basic metabolic panel (BMP), and starting direct breastfeeding as needed. Later that evening, an examination at that time revealed a healthy-appearing infant with a strong cry, clear lungs, regular heart sounds, a soft abdomen, strong pulses, and normal reflexes. The temperature was within normal limits.

Over the NICU stay, the baby passed meconium within 24 hours of life without the need for rectal stimulation. He continued stooling and urinating well. CBC showed elevated WBC (white blood cell) levels, with normal RBC (red blood cell), hemoglobin, hematocrit, and platelets (Table 1). CRP was slightly elevated, but blood cultures remained negative after 72 hours. On the third day, the baby was discharged with instructions to follow up with his general pediatrician in one to two days.

**Table 1:** NICU admission lab results BUN: Blood Urea Nitrogen; WBC: White Blood Cells; RBC: Red Blood Cells; Hgb: Hemoglobin; Hct: Hematocrit; MCV: Mean Corpuscular Volume; MCH:Mean Corpuscular Hemoglobin; MCHC: Mean Corpuscular Hemoglobin Concentration; RDW-SD: Red Cell Distribution Width - Standard Deviation; MPV: Mean Platelet Volume; CRP: C-Reactive Protein; H:High Value Flag; L: Low Value Flag

Lab Test	Reference Values	Day 1	Day 2
Sodium (mmol/L)	135-145	141	-
Potassium (mmol/L)	3.7-5.9	5	-
Chloride (mmol/L)	96-106	106	-
BUN (mg/dL)	4-12	9	-
Creatinine (mg/dL)	0.3-1.0	0.84	-
Glucose (mg/dL)	40-60 (<24 hrs); 50-100 (>24 hrs)	79 (H)	-
Calcium (mg/dL)	7.5-12.0	9.5	-
Total bilirubin (mg/dL)	1.0-12.0	-	-
Lactic acid (mmol/L)	0.5-2.5	-	-
Magnesium (mg/dL)	1.5-2.3	-	-
Procalcitonin (ng/mL)	0.1-0.5	-	-
WBC (10^3^/µL)	9.0-30.0	22.71	24.68
RBC (10^6^/µL)	4.0-6.6	5.93	5.6
Hgb (g/dL)	14.0-24.0	20.8	19.6
Hct (%)	42-65	58.8	56.4
MCV (fL)	95-121	99	101
MCH (pg/cell)	29-37	35.1	35
MCHC (g/dL)	30-36	35.4	34.8
RDW-SD (fL)	39-55	58.2 (H)	57.4 (H)
Platelets (10^3^/µL)	150-450	219	257
MPV (fL)	7.0-11.0	10.4	11
Platelet estimate (qualitative)	Normal	Normal	Normal
CRP (mg/L)	<3	3.2 (H)	-

At follow-up at nine days of age, the baby presented with no complaints, and the mother stated that she was feeding Enfamil formula appropriately. Three days later, at 12 days of age, the baby returned to the office due to not having stooled for three days. Additionally, the mother noted decreased appetite, reduced urinary output, and frequent hiccups but denied any other symptoms. The baby appeared listless on physical exam with signs of jaundice. The patient was referred to the emergency department (ED) to rule out sepsis.

**Figure 1:**
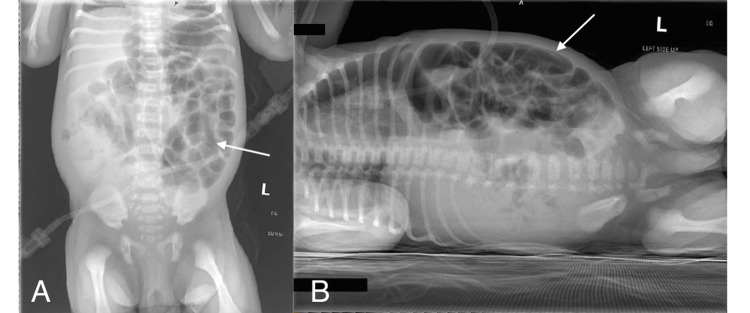
X-ray abdomen taken at first admission (A) *Supine positioning.* The arrow points to multiple air-filled small bowel loops; (B) *Right lateral decubitus positioning.* The arrow points to multiple air-filled small bowel loops. Overall findings: Multiple air-filled small bowel loops. No air-fluid levels. Stool within the colon. No evidence of pneumoperitoneum. No organomegaly. Osseous structures are within normal limits for age. Overall impression: Nonspecific bowel gas pattern.

Upon presentation to the ED that same day, the baby was readmitted due to his decreased energy, poor oral intake, decreased wet diapers, and jaundice. Lab results showed elevated bilirubin levels, and phototherapy was initiated (Table 2). Infectious disease consultation was made to rule out sepsis and meningitis. Multiple lumbar puncture attempts yielded minimal fluid collection. Antibiotics (ampicillin and ceftazidime) were started. Diagnostic imaging was obtained. Abdominal ultrasound showed no acute abnormalities or pyloric stenosis. Abdominal X-ray indicated a nonspecific bowel gas pattern (Figures 1A, 1B). The patient continued on antibiotics, and multiple lumbar puncture attempts were still unsuccessful in collecting enough fluid for studies. Over the hospital course, the baby showed improved feeding and activity levels, and antibiotics were continued pending culture results. The baby was clinically stable, afebrile, feeding, stooling, and urinating well. Cultures were negative after 72 hours, and he was discharged with instructions to follow up with his pediatrician and an infectious disease specialist. At follow-up with the pediatrician the next day, the baby was at baseline.

**Table 2:** Initial hospital admission lab results BUN: Blood Urea Nitrogen; WBC: White Blood Cells; RBC: Red Blood Cells; Hgb: Hemoglobin; Hct: Hematocrit; MCV: Mean Corpuscular Volume; MCH: Mean Corpuscular Hemoglobin; MCHC: Mean Corpuscular Hemoglobin Concentration; RDW-SD: Red Cell Distribution Width - Standard Deviation; MPV: Mean Platelet Volume; CRP: C-Reactive Protein; H: High Value Flag; L: Low Value Flag

Lab Test	Reference Values	Day 1	Day 3	Day 4	Day 6
Sodium (mmol/L)	135-145	140	138	-	-
Potassium (mmol/L)	3.4-4.5	5.6 (H)	4.4	-	-
Chloride (mmol/L)	96-106	109 (H)	110 (H)	-	-
BUN (mg/dL)	9-23	6 (L)	7 (L)	-	-
Creatinine (mg/dL)	0.7-1.3	0.32 (L)	<0.20 (L)	-	-
Glucose (mg/dL)	50-80	96 (H)	97 (H)	-	-
Calcium (mg/dL)	7.5-12.0	9.8	10.1	-	-
Total bilirubin (mg/dL)	1.0-12.0	-	10.1 (H)	-	-
Lactic acid (mmol/L)	0.5-2.5	-	-	-	-
Magnesium (mg/dL)	1.5-2.3	-	-	-	-
Procalcitonin (ng/mL)	0.1-0.5	-	-	0.58 (H)	0.13
WBC (10^3^/µL)	9.0-30.0	16.52	13.58	9.14	14.82
RBC (10^6^/µL)	4.0-6.6	5.81	4.73	4.49	4.63
Hgb (g/dL)	14.0-24.0	19.9	16.1	15.4	15.5
Hct (%)	42-65	56.2	46.5	44.9	43.9
MCV (fL)	95-121	97	98	100	95
MCH (pg/cell)	29-37	34.3	34	34.3	33.5
MCHC (g/dL)	30-36	35.4	34.6	34.3	35.3
RDW-SD (fL)	39-55	53.1 (H)	54.6 (H)	54.7 (H)	50.5
Platelets (10^3^/µL)	150-450	315	338	372	445 (H)
MPV (fL)	7.0-11.0	11.9	12.2	11.5	11.1
Platelet estimate (qualitative)	Normal	Normal	Normal	Normal	Normal
CRP (mg/L)	<3	1.94	-	53.46 (H)	11.27 (H)

At 24 days, the baby returned to the pediatrician due to one day of severe and worsening diarrhea. Oral intake remained normal, and the baby was stable with maintained weight. Stool heme testing was positive. On physical exam, the baby had hyperactive bowel sounds. A lactose-free diet was encouraged for suspected milk protein allergy. On follow-up five days later, diarrhea persisted with loss of appetite. Allergic colitis was suspected, leading to the infant being placed on a hypoallergenic formula, resulting in symptom resolution.

**Figure 2:**
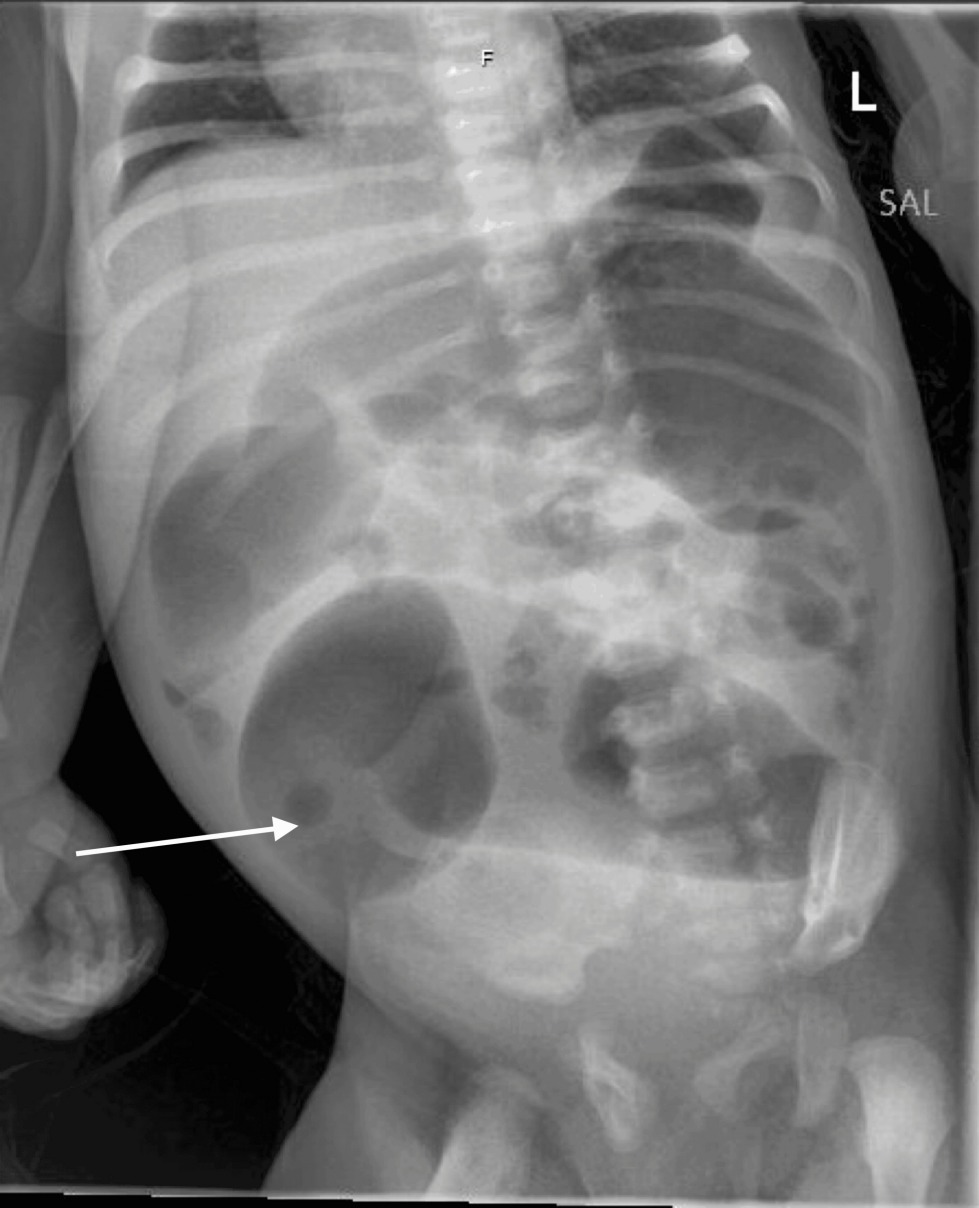
One-view X-ray abdomen taken on day 1 of the second admission The arrow points to a prominent air-filled loop of the bowel. Findings: A prominent air-filled loop of the bowel measuring 4.5 cm in diameter within the lower abdomen without evidence of rectal air. Loops of normal caliber small bowel are seen within the mid-abdomen. No evidence of bowel obstruction. The visualized osseous structures are normal. Overall impression: The prominent loop of the bowel likely represents the distal colon, which suggests a possible Hirschsprung's disease. There is no evidence of obstruction.

At four weeks old, the baby presented to the ED with a fever of two days recorded at 102°F and loose stool. Lab results showed elevated WBC, decreased hemoglobin and hematocrit, and elevated CRP levels (Table 3). An abdominal X-ray was obtained and revealed a prominent air-filled bowel loop suggestive of HD (Figure 2). A renal ultrasound was normal except for nonspecific debris in the bladder.

**Table 3:** Second admission lab results BUN: Blood Urea Nitrogen; WBC: White Blood Cells; RBC: Red Blood Cells; Hgb: Hemoglobin; Hct: Hematocrit; MCV: Mean Corpuscular Volume; MCH: Mean Corpuscular Hemoglobin; MCHC: Mean Corpuscular Hemoglobin Concentration; RDW-SD: Red Cell Distribution Width - Standard Deviation; MPV: Mean Platelet Volume; CRP: C-Reactive Protein; H: High Value Flag; L: Low Value Flag

Lab Test	Reference Values	Day 1	Day 2	Day 3	Day 4	Day 5	Day 7	Day 8	Day 12	Day 13	Day 18	Day 20	Day 22
Sodium (mmol/L)	135-145	136	139	144	139	-	138	-	-	140	139	144	139
Potassium (mmol/L)	3.4-4.5	5.0 (H)	3.9	3.2 (L)	3.6	-	4.2	-	-	4.8 (H)	4.6 (H)	4.1	4.5
Chloride (mmol/L)	96-106	102	111 (H)	112 (H)	111 (H)	-	109 (H)	-	-	111 (H)	112 (H)	119 (H)	113 (H)
BUN (mg/dL)	9-23	16	12	11	8 (L)	-	7 (L)	-	-	11	10	<6 (L)	<6 (L)
Creatinine (mg/dL)	0.7-1.3	0.32 (L)	<0.20 (L)	<0.20 (L)	<0.20 (L)	-	<0.20 (L)	-	-	<0.20 (L)	<0.20 (L)	<0.20 (L)	<0.20 (L)
Glucose (mg/dL)	50-80	98	77	91	73	-	84	-	-	81	89	82	76
Calcium (mg/dL)	7.5-12.0	9.8	9.6	9.7	8.3	-	8.9	-	-	9.5	9.6	9.4	9.9
Total bilirubin (mg/dL)	1.0-12.0	0.9	0.7	0.8	-	-	0.3	-	-	0.3	<0.2 (L)	-	-
Lactic acid (mmol/L)	0.5-2.5	-	2.0 (H)	-	-	2.3 (H)	0.8	-	-	-	-	-	-
Magnesium (mg/dL)	1.5-2.3	-	-	-	-	-	-	-	-	-	-	2.2	
Procalcitonin (ng/mL)	0.1-0.5	6.01 (H)	17.38 (H)	-	-	6.25 (H)	-	-	-	<0.09	-	-	-
WBC (10^3^/µL)	9.0-30.0	28.46 (H)	25.89 (H)	-	27.13 (H)	15	21.15 (H)	20.42	13.96	14.13	8.01	15.43	12.24
RBC (10^6^/µL)	4.0-6.6	3.17	2.74 (L)	-	2.37 (L)	2.35 (L)	2.17 (L)	3.25	3.44	3.42	2.93 (L)	2.59 (L)	4.19
Hgb (g/dL)	14.0-24.0	10.3	8.7 (L)	-	7.6 (L)	7.7 (L)	7.0 (L)	10.1	10.5	10.6	9.1 (L)	8.0 (L)	13.1
Hct (%)	42-65	30.4 (L)	26.7 (L)	-	22.3 (L)	22.3 (L)	21.4 (L)	29.2 (L)	32.4	31.8	27.0 (L)	23.9 (L)	36.7
MCV (fL)	95-121	96	97	-	94	95	99	90	94	93	92	92	88
MCH (pg/cell)	29-37	32.5	31.8	-	32.1	32.8	32.3	31.1	30.5	31	31.1	30.9	31.3
MCHC (g/dL)	30-36	33.9	32.6	-	34.1	34.5	32.7	34.6	32.4	33.3	33.7	33.5	35.7
RDW-SD (fL)	39-55	53.4 (H)	54.6 (H)	-	54.5 (H)	54.9 (H)	56.5 (H)	58.2 (H)	67.6 (H)	64.8 (H)	61.1 (H)	60.1 (H)	50.1
Platelets (10^3^/µL)	150-450	604 (H)	484 (H)	-	-	432 (H)	490 (H)	231	380	272	423 (H)	401 (H)	506 (H)
MPV (fL)	7.0-11.0	10	10.1	-	11.4	10.5	10.8	11.6	11.4	11.7	11.6	11.3	10.4
Platelet estimate (qualitative)	Normal	Increased!	Increased!	-	Normal	-	Normal	Normal	Normal	Normal	-	-	-
CRP (mg/L)	<3	>200.00 (H)	>200.00 (H)	-	-	65.14 (H)	15.29 (H)	-	-	3.65 (H)	-	-	-

**Figure 3:**
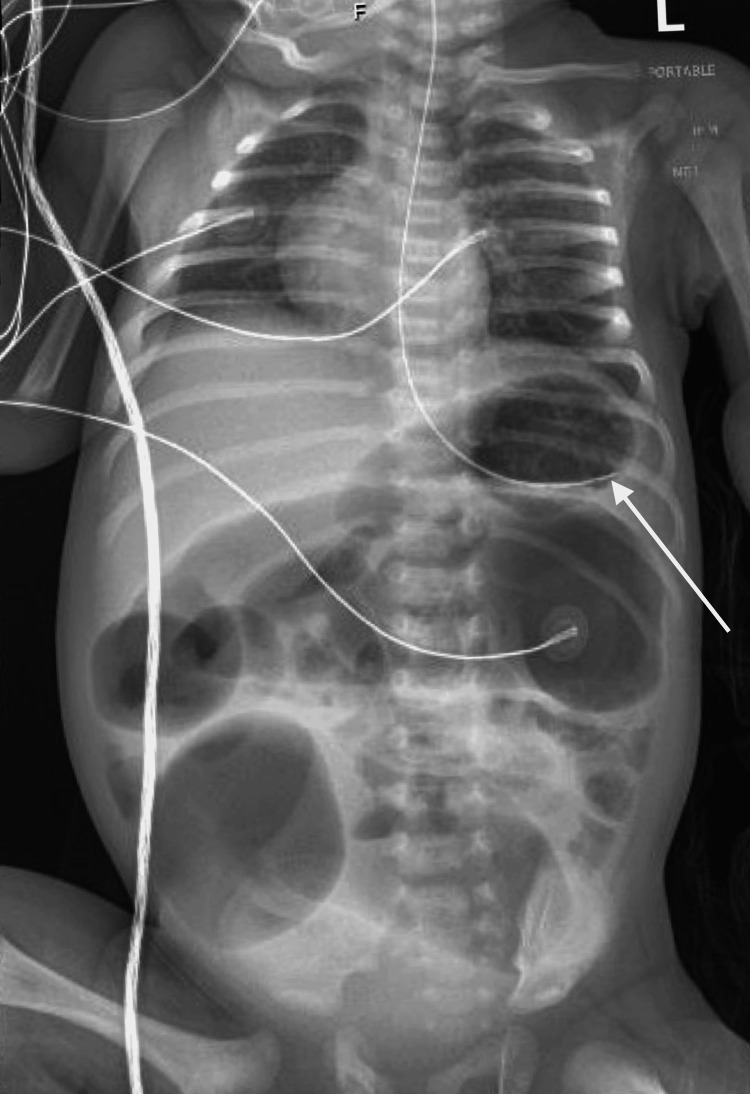
One-view X-ray abdomen taken on day 3 of the second admission The arrow points to the tip of the enteric tube inside the stomach. Overall impression: Interval placement of an enteric tube that follows the expected course of the esophagus. The tip overlies the stomach bubble.

Rectal suction biopsy was obtained and confirmed HD. The baby was diagnosed with enterocolitis secondary to HD, and initial treatments included intravenous metronidazole, piperacillin-tazobactam, and ampicillin, with twice-daily rectal washes. His condition improved with abdominal decompression (Figure 3) and feeding hypoallergenic formula. Over the next few days, the patient showed further improvement with stable vitals, normal stools, and good feeding. Infection markers decreased, and surgery was planned for definitive treatment.

At seven weeks of age, the baby underwent a laparoscopic-assisted primary endorectal pull-through procedure for HD. The procedure involved leveling colonic biopsies with a frozen section to establish a rectosigmoid transition. The resected rectosigmoid was further confirmed to have absent ganglion cells at the lower rectal end and abundant ganglion cells at the colonic anastomotic line.

Upon transfer to PICU, the baby began to have regular bowel movements and demonstrated good urine output. H/H levels were slightly low, requiring 90 cc of transfused packed RBCs (Table 3). During hospitalization, he was started on Pedialyte at 5 mL, which was gradually increased to 15 mL every three hours, before transitioning to Alimentum at the same rate. By the evening of postop day three, the baby was upgraded to full feeds of 3-4 oz every four hours. An abundant zinc oxide paste was applied frequently to the perineum, and the patient received metronidazole and piperacillin-tazobactam alongside intravenous acetaminophen for pain management. The pediatric surgeon approved discharge on postop day four. The patient was discharged on metronidazole and scheduled for follow-up appointments with his pediatrician and surgeon, eventually making a complete recovery.

## Discussion

This case depicts an unusual initial presentation of HD, primarily because the infant appeared to be stooling normally during the majority of the first few weeks of life. Typically, HD generally presents in neonates with symptoms of bowel obstruction, such as failure to pass meconium within the first 48 hours of life, abdominal distention, and vomiting. The condition often manifests shortly after birth, making early diagnosis crucial to avoid complications such as enterocolitis [8]. The unusual aspect of this case is the near consistent, normal stooling, which typically delays the suspicion of HD until later in infancy [9].

Given that the infant was stooling normally during the initial NICU admission, HD was not initially suspected, and abdominal imaging was not performed. The normal bowel movements initially led to a focus on sepsis, and routine labs, as well as blood cultures, seemed sufficient at that time. In cases where neonates are suspected of early sepsis and are being assessed solely for perinatal risk factors, performing a lumbar puncture may not be necessary and, therefore, was not performed [10]. Despite negative blood cultures, the inability to isolate a microbial pathogen does not exclude sepsis [11]. Due to the baby stabilizing upon treatment with antibiotics, he was discharged home and had follow-ups in place.

However, the recurrence of symptoms that required the first admission, including fever of unknown origin and signs of infection, as well as new-onset jaundice, lethargy, and no stool within three days, prompted further diagnostic imaging and labs. The first abdominal X-ray, as seen in Figures 1A, 1B, showed a nonspecific bowel gas pattern, which did not raise suspicion for any gastrointestinal (GI) concern [12]. The abdominal ultrasound was also unremarkable, so no further workup was done to rule out GI causes. Multiple lumbar punctures were also attempted to rule out meningitis, but due to the small amount of spinal fluid being collected, the results were inconclusive. Over this hospital course, the baby responded well to antibiotics once again and clinically improved. A key aspect of the presentation was that the baby was stooling and urinating well over the course of the hospital stay as he improved. With negative blood cultures, discharge home with follow-ups was reasonable as culture-negative sepsis or meningitis were the two leading differentials [13].

When the baby was admitted for the second time, an X-ray of the abdomen was repeated due to unresolved diarrhea and revealed a prominent air-filled bowel loop suggestive of HD [14]. The presence of stooling is unexpected in a case of possible HD, but a suction biopsy was conducted and confirmed the diagnosis [9]. It had finally been concluded that, since birth, the baby's fever was due to enterocolitis secondary to HD.

As the baby had none of the typical red flag symptoms that pointed to HD and the initial abdominal X-ray was unremarkable, enterocolitis was not suspected early on. Also, with inconclusive results from the attempted lumbar punctures, it was impossible to confidently rule out meningitis as an initial cause for fever. The baby's GI symptoms, most notably heme-positive diarrhea, improved with the supplementation of hypoallergenic formula. This led the physicians to suspect a more transient, resolvable issue, such as allergic colitis, rather than a chronic, congenital abnormality. It is now evident that because the underlying disorder strayed from the expected presentation, it allowed other plausible but unconfirmed diagnoses to rise in the differential. Here, we show this atypical presentation to enhance the depth of the literature and allow for improved clinical decision-making.

## Conclusions

This case emphasizes the importance of considering HD in the differential diagnosis of neonates and infants with recurrent sepsis-like presentations. It illustrates how the disease can present in an atypical fashion, complicating timely diagnosis and management. Clinicians should maintain a high index of suspicion and employ a combination of clinical, radiological, and histological assessments to accurately diagnose and effectively treat HD, even in the face of unusual presentations.
